# Pre‐operative three‐dimensional face scans for predicting difficult facemask ventilation: a prospective development study

**DOI:** 10.1111/anae.70261

**Published:** 2026-06-05

**Authors:** Viktor A. Wünsch, Hannes Bommes, Sofia Germer, Milan Piltan, Philipp Breitfeld, Sarah Latus, Linda Krause, Christian Zöllner, Christopher Gundler, Martin Petzoldt

**Affiliations:** ^1^ Department of Anaesthesiology, Centre for Anaesthesiology and Intensive Care Medicine University Medical Centre Hamburg‐Eppendorf Hamburg Germany; ^2^ Institute for Applied Medical Informatics University Medical Centre Hamburg‐Eppendorf Hamburg Germany; ^3^ Institute of Medical Technology and Intelligent Systems Hamburg University of Technology Hamburg Germany; ^4^ Institute of Medical Biometry and Epidemiology University Medical Centre Hamburg‐Eppendorf Hamburg Germany

**Keywords:** airway management, facial recognition, respiratory system, ventilation

## Abstract

**Introduction:**

Facemask ventilation is a key airway management skill but predicting difficulty can be challenging. Pre‐operative three‐dimensional face scanning may have diagnostic value. We aimed to identify interpretable facial shape features and to quantify their value for predicting difficult facemask ventilation.

**Methods:**

In this prospective observational single‐centre study, pre‐operative three‐dimensional face scans were obtained, and a structured airway assessment was performed on patients undergoing ear, nose and throat or maxillofacial surgery. The primary outcome was difficult facemask ventilation documented as an alert in the patient health record. After postprocessing, three‐dimensional face scans were fitted to an established, non‐clinical facial model to identify interpretable shape coefficients. The area under the receiver operating characteristic (AUROC) curve for the DIFFMASK score was calculated before and after enrichment with three facial shape features and the added diagnostic value was assessed using likelihood ratios.

**Results:**

Data from 398 patients were analysed. The optimism‐corrected AUROC was 0.73 (95%CI 0.65–0.80) for the DIFFMASK score and 0.74 (95%CI 0.66–0.82) for selected facial shape features. Enrichment of the DIFFMASK score with three facial shape features improved goodness of model fit (p = 0.002) and achieved an optimism‐corrected AUROC of 0.76 (95%CI 0.68–0.82). Generated face meshes with superimposed colour mapping revealed that morphological features of the nose, lower mandible, neck region and facial convexity were most predictive of difficult facemask ventilation.

**Discussion:**

Pre‐operative three‐dimensional face scans predicted difficult facemask ventilation at least as well as the DIFFMASK score. Integrating the features of three selected facial shapes enriched the DIFFMASK score and improved its diagnostic value. Digital phenotyping can complement traditional clinical assessment.

## Plain Language Summary

**What we did:**

Researchers studied 398 patients having head and neck surgery. Before surgery, they used a special 3D scanner to record and study each patient's face and carried out normal airway checks. After the patients were given anaesthesia, doctors used a facemask to ventilate them. Doctors recorded when facemask ventilation was difficult. The researchers then looked for facial features in the 3D scans that might help predict when facemask ventilation would be difficult.

**Why did we do it:**

Ventilation with a facemask is an important part of anaesthesia, but it can sometimes be difficult. Doctors wanted to know if 3D face scans could help spot patients in advance before anaesthesia who may develop difficulties. This could help doctors prepare and improve patient safety.

**What we found:**

The 3D face scans worked at least as well as the usual clinical scoring system at predicting difficult facemask ventilation. When the researchers combined the face scan information with the usual clinical scoring system, the prediction became even better. Certain facial features, such as the shape of the nose, jaw, neck and face, were linked with more difficult facemask ventilation. The study showed that 3D face scanning could be a useful extra tool for doctors when planning anaesthetic care.

## Introduction

Facemask ventilation is an essential airway management skill for the prevention and treatment of hypoxaemia [1, 2]. Difficult facemask ventilation can contribute to airway‐related adverse events [3]. The prediction of difficult facemask ventilation is based largely on clinical assessment [4, 5, 6]. Physical airway examination tests such as the modified Mallampati test, thyromental distance and inter‐incisor distance have low sensitivity (17%, 13% and 6%, respectively) and reasonable specificity (90%, 94% and 91%, respectively) for predicting difficult facemask ventilation [7]. DIFFMASK is a recognised clinical risk score for predicting difficult facemask ventilation during general anaesthesia. The score originated from a model development study with 46,804 patients and comprises 10 predictors: age; sex; BMI; previous difficult tracheal intubation; thyromental distance; Mallampati score; full beard; snoring; sleep apnoea; and neck radiation changes [8]. The DIFFMASK score had a sensitivity of 85% and specificity of 59% for predicting difficult facemask ventilation in the original development study [8].

It remains uncertain which morphological facial features are best suited to predicting difficult facemask ventilation. Non‐invasive diagnostic approaches such as pre‐operative analysis of vocal [9, 10] or facial [11] characteristics have been proposed to enhance clinical assessments. These diagnostic procedures offer the potential to identify subtle anatomical or functional variations that may not be detected during conventional clinical examination. However, their diagnostic accuracy and clinical utility require further investigation, including whether they are an alternative to physical examination or bring additional value when combined with physical examination.

We aimed to systematically identify interpretable facial shape features gathered from pre‐operative three‐dimensional face scans, and to quantify their ability to predict difficult facemask ventilation in comparison with, and in addition to, the DIFFMASK score [8].

## Methods

The single‐centre prospective observational MASCAN study was approved by the Ethics Committee of the Medical Association of Hamburg and conducted in accordance with the revised Declaration of Helsinki [12]. Findings are reported in adherence to the updated TRIPOD+AI statement [13]. Patients provided written informed consent.

Patients were assessed for study eligibility if they were aged ≥ 18 y and scheduled for general anaesthesia for elective ear, nose and throat or maxillofacial surgery that incorporated facemask ventilation and tracheal intubation. Patients were not studied if awake tracheal intubation or rapid sequence induction was indicated, or if they were pregnant or breastfeeding.

Patients underwent a structured airway assessment that included: a review of their medical conditions and previous airway management; Wilson score; Simplified Airway Risk Index; and upper lip bite test [7, 14, 15]. Transnasal videoendoscopy was performed, if appropriate [16, 17]. The need for awake tracheal intubation was evaluated using the Expect‐It decision‐making tool [18].

We selected clinical predictor variables for difficult facemask ventilation based on clinical considerations and literature review, comprising: sociodemographic data; medical history; bedside airway assessment tests; and physical examination [4, 5, 6, 7, 8, 19, 20, 21, 22, 23] (Tables 1 and 2). These variables were assessed using a structured case report form by two researchers who were not involved in decision‐making regarding patient care or airway management. A single‐use measuring tape with a millimetre scale was used to measure: inter‐incisor distance (between the upper and lower incisors in the midline with maximum mouth opening) [24]; thyromental distance (from the front of the thyroid cartilage to the chin with the head fully extended); and sternomental distance (from the sternal notch to the chin). The DIFFMASK score was calculated using the relevant variables.

**Table 1:** Characteristics of the study cohort. Values are median (IQR [range]) or number (proportion).

	All patients	Easy facemask ventilation*	Difficult facemask ventilation*
	(n = 398)	(n = 355)	(n = 43)
**Patient characteristics**			
Age; y	48 (32–63 [18–88])	47 (31–62 [18–88])	53 (37–80 [26–80])
BMI; kg.m^‐2^	25 (23–28 [16–51])	25 (22–28 [16–51])	29 (25–34 [20–51])
Sex; female	181 (45.5%)	167 (47.0%)	14 (32.6%)
ASA physical status			
1	93 (23.4%)	87 (24.5%)	6 (14.0%)
2	223 (56.0%)	200 (56.3%)	23 (53.5%)
3	81 (20.3%)	68 (19.2%)	13 (30.2%)
4	1 (0.3%)	‐	1 (2.3%)
**Type of surgery**			
Dentoalveolar	29 (7.3%)	25 (7.0%)	4 (9.3%)
Ear, nose, sinus	161 (40.5%)	143 (40.3%)	18 (41.8%)
Endocrine/exocrine glands	24 (6.0%)	22 (6.2%)	2 (4.7%)
Mandibular	39 (9.8%)	35 (9.9%)	4 (9.3%)
Microlaryngoscopy	24 (6.0%)	21 (5.9%)	3 (7.0%)
Neck and maxillofacial	58 (14.6%)	51 (14.4%)	7 (16.2%)
Pharyngolaryngeal	53 (13.3%)	50 (14.0%)	3 (7.0%)
Other	10 (2.5%)	8 (2.3%)	2 (4.7%)
**Airway operators**			
Age; y	30 (27–34 [25–55])	30 (27–33 [25–55])	31 (28–34 [25–54])
Professional work experience in months	30 (4–60 [1–286])	30 (4–59 [1–286])	48 (4–61 [1–286])
**Outcome measures**			
Difficult facemask ventilation	43 (10.8%)	‐	43 (100%)
Impossible facemask ventilation	3 (0.8%)	‐	3 (7.0%)
Airway related adverse events	10 (2.5%)	9 (2.6%)	1 (2.3%)

* Refers to whether the primary study outcome ‘difficult facemask ventilation’ was achieved or not (easy facemask ventilation).

**Table 2:** Clinical predictor variables for difficult facemask ventilation in the study cohort. Values are number (proportion) or median (IQR [range]).

	All patients	Easy facemask ventilation*	Difficult facemask ventilation*
	(n = 398)	(n = 355)	(n = 43)
Mallampati score			
1	175 (44.0%)	163 (45.9%)	12 (27.9%)
2	101 (25.4%)	91 (25.7%)	10 (23.3%)
3	68 (17.1%)	58 (16.3%)	10 (23.3%)
4	54 (13.5%)	43 (12.1%)	11 (25.5%)
Upper lip bite test			
1	272 (68.4%)	242 (68.2%)	30 (69.8%)
2	94 (23.6%)	85 (23.9%)	9 (20.9%)
3	32 (8.0%)	28 (7.9%)	4 (9.3%)
Beard			
Stubble	61 (15.3%)	53 (14.9%)	8 (18.6%)
Full beard	53 (13.3%)	40 (11.3%)	13 (30.2%)
Head and neck dysmorphia	53 (13.3%)	41 (11.5%)	12 (27.9)
Inter‐incisor distance; cm	4.7 (4.2–5.3 [1.5–9.0])	4.8 (4.2–5.3 [1.5–9.0])	4.5 (4.0–5.1 [1.5–7.0])
Sternomental distance; cm	18 (17–20 [12–25])	18 (17–20 [13–25])	18 (15–20 [12–23])
Thyromental distance; cm	11 (10–12 [7–15])	11 (10–12 [7–15])	10 (9–11 [7–13])
Snoring	190 (47.7%)	157 (44.2%)	33 (76.7%)
Sleep apnoea	31 (7.8%)	25 (7.0%)	6 (14.0%)
Neck radiation skin changes	15 (3.8%)	12 (3.4%)	3 (7.0%)
Retrognathia			
Moderate	21 (5.3%)	20 (5.6%)	1 (2.3%)
Severe	20 (5.0%)	18 (5.1%)	2 (4.7%)
Prominent incisors			
Moderate	54 (13.6%)	52 (14.6%)	2 (4.7%)
Severe	18 (4.5%)	17 (4.8%)	1 (2.3%)
Dental prothesis			
Partial denture	19 (4.8%)	17 (4.8%)	2 (4.7%)
Complete upper or lower denture	20 (5.0%)	14 (3.9%)	6 (13.9%)
Complete upper and lower denture	17 (4.3%)	15 (4.2%)	2 (4.7%)
Neck movement			
< 70°	5 (1.3%)	3 (0.9%)	2 (4.7%)
71–80°	22 (5.5%)	16 (4.5%)	6 (13.9%)
81–90°	95 (23.9%)	86 (24.2%)	9 (20.9%)
> 90°	276 (69.3%)	250 (70.4%)	26 (60.5%)
Previous difficult tracheal intubation**			
Questionable	6 (1.5%)	5 (1.4%)	1 (2.3%)
Yes	3 (0.8%)	1 (0.3%)	2 (4.7%)

* Refers to whether the primary study outcome ‘difficult facemask ventilation’ was achieved or not (easy facemask ventilation).

** As defined in the Simplified Airway Risk Index [14].

All three‐dimensional face scans were performed in the same examination room within a pre‐assessment clinic with identical lighting and setup by the two researchers mentioned above. The three‐dimensional facial scans were acquired with a structured‐light scanner (Artec EVA, Artec 3D, Senningerberg, Luxembourg) which is validated for medical use, and using the associated software (Artec Studio 17 Professional) [25]. Scans were obtained at an optimal distance of 52–88 cm, over a duration < 60 s, with patients seated upright with neutral head position, eyes and mouth closed (no clenching of teeth), hair tied back and accessories removed. Scans were checked manually for artefacts and processed on a dedicated workstation. Further details regarding the three‐dimensional scanning process are in online Supporting Information Appendix S1.

In the operating theatre, the anaesthetist attempted facemask ventilation using a silicone mask (Armstrong Medical, Coleraine, UK) after the loss of eyelid reflex, delivering a fraction of inspired oxygen of 1.0 at 15 l.min^‐1^ via a mechanical ventilator (Perseus^®^ A500, Draeger Medical, Lübeck, Germany) with standardised settings (inspiratory pressure 15 cmH_2_O, positive end‐expiratory pressure 0 cmH_2_O, respiratory rate 10.min^‐1^, I:E ratio 1:2) [26, 27, 28]. Facemask ventilation was evaluated after induction of anaesthesia and before administration of a neuromuscular blocking drug. The airway manager could adjust head and neck position and use rescue manoeuvres such as two‐handed grip; oral airway; jaw thrust; converting to manual ventilation; or allowing a senior consultant anaesthetist to take over. Details regarding anaesthesia induction, facemask ventilation and airway management are in online Supporting Information Appendix S1.

Outcome measures were assessed systematically in the operating theatre by an independent observer not involved in patient care and using standardised case report forms. The primary outcome measure was difficult facemask ventilation, defined as an alert documented in the patient health records by the airway operator after the difficulty was encountered. This alert was issued based on the clinical consideration of the airway operator within routine practice and not on predefined criteria. Secondary outcomes were impossible facemask ventilation and airway‐related adverse events, defined as one or more of: airway or oral trauma including bleeding and dental injury; glottic swelling; hypotension (systolic blood pressure < 70 mmHg); hypoxaemia (SpO_2_ ≤ 93%); and laryngospasm. Impossible facemask ventilation was defined as absence of end‐tidal carbon dioxide measurement and lack of perceptible chest wall movement despite airway adjuvants, assistance of additional personnel and neuromuscular blockade [19, 22].

A prevalence of 11% for difficult facemask ventilation was anticipated based on findings reported in a Cochrane review [7] and institutional experience [16, 18, 29, 30]. We used log likelihood‐ratio tests to assess the incremental benefit of adding facial features to recognised clinical parameters. We conducted a sample size justification considering two logistic regressions, with three degrees of freedom [11], α = 0.01, and a power of 80%. Based on these assumptions, a sample size of 345 patients was adequate to detect a mean log‐likelihood difference per patient of 0.03.

**Figure 1:**
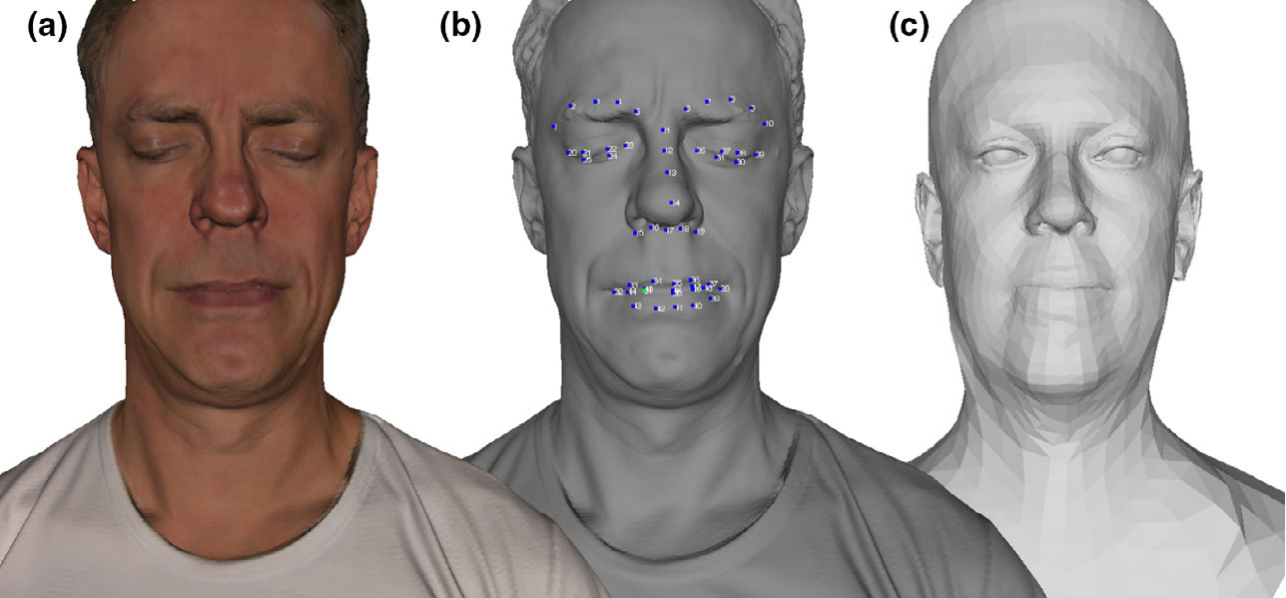
Overview of the data preprocessing pipeline for fitting facial scans to the FLAME model to enable subsequent analysis and model training: (a) raw mesh after manual cleaning; (b) landmarks after adjustment for the expected landmarks of the fitting procedure; and (c) generated mesh from the FLAME model given the identity coefficients.

We designed a data processing pipeline for generating three‐dimensional models of the face of each patient. After denoising and smoothing, we extracted 73 facial landmarks automatically using a pretrained convolutional neural network [31] and fitted each scan to ‘prototypical’ heads derived from the Faces Learned with an Articulated Model and Expressions (FLAME) model [32]. This is a statistical 3D head model that represents facial shape, pose and expression within an anatomically grounded parameterisation, trained on a dataset of more than 33,000 faces spanning diverse populations. After fitting, we used its first 100 shape coefficients to represent the global facial morphology (online Supporting Information Appendix S1). The pipeline for converting raw three‐dimensional face scans into interpretable coefficients is shown in Figure 1. Based on the interpretable FLAME model, we kept all identity‐related coefficients constant, systematically varied only the three coefficients identified as most relevant by logistic regression, and generated the corresponding three‐dimensional meshes. We then computed the average faces of patients identified as having lower and higher risk of difficult facemask ventilation, stratified by sex. The resulting differences between meshes were visualised with colour mapping on the average face for each sex.

To assess whether interpretable facial features improve prediction beyond standard clinical variables, we used adaptive best subset selection to identify the most predictive facial shape descriptors from 100 available shape coefficients [33]. First, we evaluated the predictive performance of facial shape features only. Using the ‘adaptive best subset’ algorithm, we selected 10 shape coefficients, analogous to the DIFFMASK score. Second, we enriched the original DIFFMASK score with three facial shape features selected through the same algorithm. We then compared nested logistic regression models (DIFFMASK alone vs. enriched with facial features) by calculating likelihood ratios and evaluating the discriminative power via optimism‐corrected AUROC (95%CI) using 1000 bootstrapped samples (Harrell's method [34]).

Statistical significance was set at p < 0.01. Continuous variables were assessed for normality via histograms. Analyses were conducted in Python 3.12 (Python Software Foundation, Beaverton, OR, USA), R 4.3.1 (R Foundation for Statistical Computing, Vienna, Austria) and SPSS 27 (IBM Inc., Armonk, NY, USA). Additional information regarding statistical analysis is in online Supporting Information Appendix S1.

## Results

**Figure 2:**
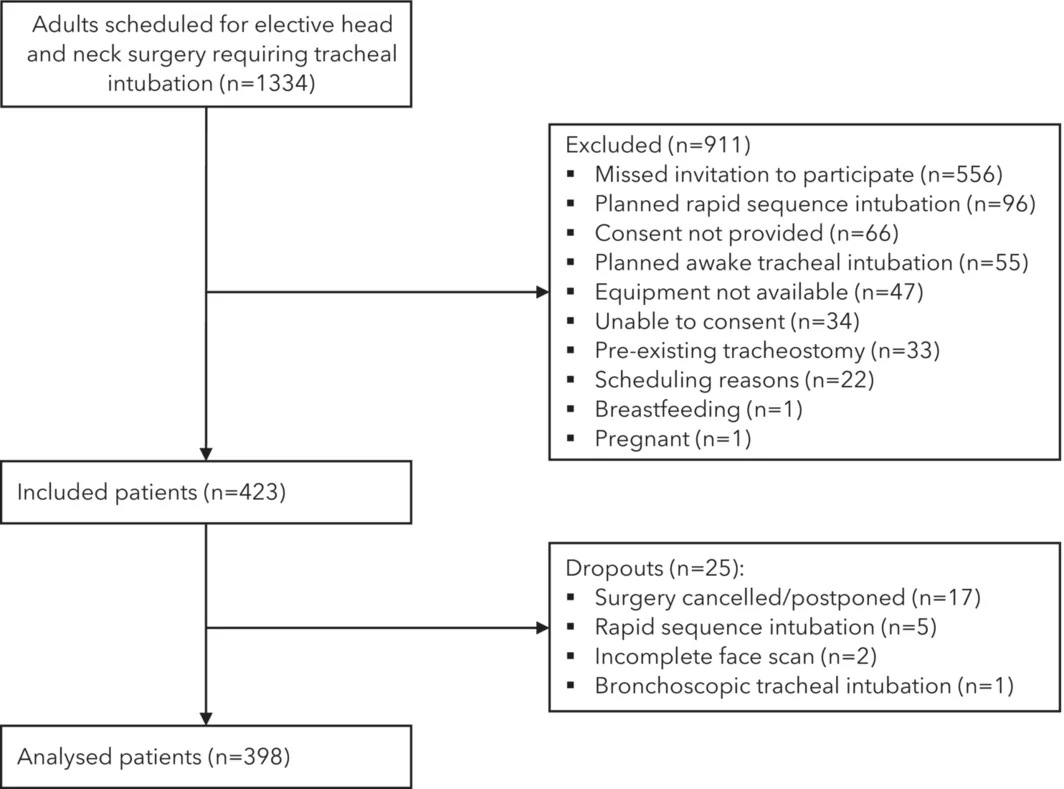
Enrolment and study flow of the MASCAN study.

Between November 2022 and May 2023, we assessed 1334 patients for eligibility and included 423 in the MASCAN study, with 398 contributing data to this analysis (Fig. 2 and Table 1). Airway operators had a median (IQR [range]) age of 30 (27–34 [25–55]) y and professional experience of 30 (4–60 [1–286]) months. Difficult facemask ventilation occurred in 43 (10.8%) patients and impossible facemask ventilation in 3 (0.8%) patients. Airway‐related adverse events were observed in 10 (2.5%) patients. Clinical predictor variables for difficult facemask ventilation are presented in Table 2. There were no missing values in the variables required for model fitting.

The non‐optimism‐corrected AUROC of the DIFFMASK score was 0.81 in our study cohort and closely resembles the AUROC of 0.82 reported in the original DIFFMASK development study (online Supporting Information Figure S1) [8]. The optimism‐corrected AUROC was 0.73 (95%CI 0.65–0.80) (online Supporting Information Figure S2). The same AUROC was achieved when the model was not re‐fitted but calculated using the ß‐coefficients of the original DIFFMASK regression model [8], indicating general robustness of the model.

**Figure 3:**
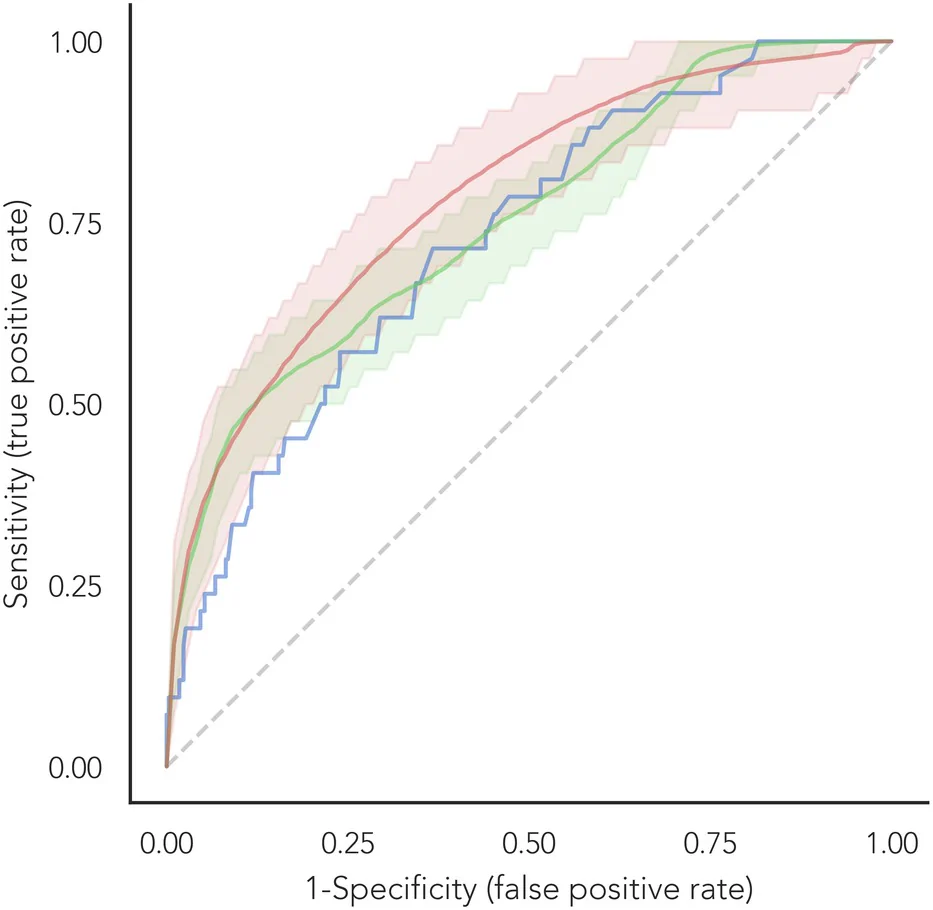
Optimism‐corrected receiver operating characteristic (ROC) curves (95%CI) of (a) the DIFFMASK score (blue line); (b) a model based on ten facial shape coefficients (green line); and (c) the DIFFMASK score enriched by three facial shape features (red line).

Isolated utilisation of the 10 most relevant facial shape features achieved an optimism‐corrected AUROC of 0.74 (95%CI 0.66–0.82) and showed similar performance to the DIFFMASK score (Fig. 3).

Supplementation of the DIFFMASK score with three facial shape features improved goodness of model fit, taking both the incremental value and added complexity into account, as determined by the likelihood‐ratio test, p = 0.002. After enrichment, the AUROC of the model increased from 0.73 (95%CI 0.65–0.80) to 0.76 (95%CI 0.68–0.82) (Fig. 3).

**Figure 4:**
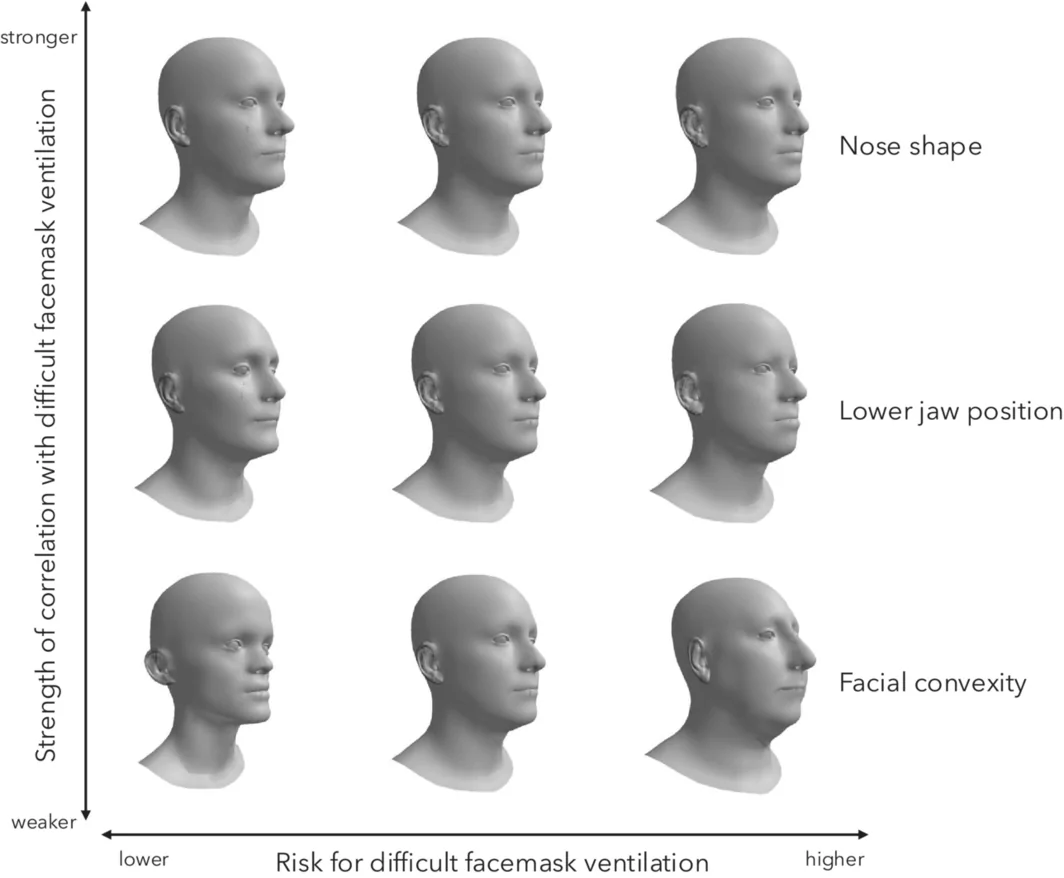
Three‐dimensional face meshes showing the features with the strongest correlation with difficult facemask ventilation in decreasing order from top to bottom. All identity‐related coefficients of the average face were kept constant while only one selected feature per row (nose shape, lower jaw position, facial convexity) being varied according to the sign of the coefficient in the logistic regression. The corresponding three‐dimensional meshes were generated based on these simulated values.

Selected morphological features that have a pronounced association with the clinical outcome are illustrated in Figure 4. Nose shape, the relative position of the lower jaw to the skull and a more convex facial profile were the most predictive features for difficult facemask ventilation with decreasing strength. Analysis of the meshes derived from the interpretable model showed that morphological features of the lower jaw and neck region were also strongly associated with difficult facemask ventilation (online Supporting Information Figure S3).

## Discussion

This prospective pilot study investigated the ability of three‐dimensional face scans and structured clinical risk assessments to predict difficult facemask ventilation. We externally validated the DIFFMASK score and replicated the performance reported in the original development study in a different surgical population. Pre‐operative three‐dimensional face scans predicted difficult facemask ventilation at least as well as the DIFFMASK score. Integrating three selected facial shape features enriched the DIFFMASK score.

The nose shape, morphological features of the lower mandible and neck region and facial convexity were most predictive characteristics for difficult facemask ventilation, probably reflecting recognised anatomical challenges. The shape of the nose can impact the seal obtained by the upper portion of the facemask, the shape of the lower mandible and its position in relation to the skull can impact the seal obtained by the lower portion of the facemask and the ability to maintain upper airway patency. Morphological features of the neck regions can influence head and neck mobility. The degree of facial convexity might also impact facemask seal. Three‐dimensional facial scans may quantify subtle morphological variations not captured readily by categorical clinical assessments, providing objective, reproducible, observer‐independent quantification that is particularly valuable in borderline cases.

Previous studies using two‐dimensional facial images and machine learning to predict difficult tracheal intubation and laryngoscopy obtained AUROC values ranging between 0.78 and 0.88 [35, 36, 37, 38, 39]. Pei et al. used three‐dimensional face scans to build a logistic regression model to predict difficult facemask ventilation, reporting an AUROC of 0.83 [11]. A composite outcome definition was used in this study, which comprised multiple clinical parameters with some low‐threshold indicators for difficult facemask ventilation, such as a tidal volume < 5 ml.kg^‐1^. Pei et al. combined the learning of the representations from the high‐dimensional face mesh with the later prediction task. The features they obtained were tailored to a Chinese population. In our study, we deliberately split the feature extraction and classification steps within our data processing. The pre‐existing FLAME as a ‘general purpose’ model provided the condition‐agnostic facial features that were then used for the prediction within the linear model. Pei et al. did not evaluate the synergistic potential of facial features and clinical assessment for predicting difficult facemask ventilation. Our study suggests that face scans capture supplemental clinical information rather than characterising features that are already identified by clinical evaluation.

Clinical risk evaluation can be time‐consuming, subjective and reliant on personal expertise. Three‐dimensional face scans enable consistent, observer‐independent data acquisition not requiring a physician. We found synergistic effects between clinical assessments and three‐dimensional facial scans, showing the potential value of combined use.

Our study has some limitations. As standards, equipment, skills and surgical populations differ between institutions, the findings of this single‐centre study should be generalised with caution. The majority of patients underwent elective head and neck surgery and were likely White European. We investigated three‐dimensional scanning equipment from one manufacturer only. This method of airway assessment has not been externally validated. The standardised and comprehensive assessment of clinical features by independent observers does not reflect standard clinical practice.

In conclusion, pre‐operative three‐dimensional face scans predict difficult facemask ventilation as effectively as the DIFFMASK score. The addition of three facial shape features improved the diagnostic value of the DIFFMASK score. Morphological features of the nose, lower mandible and neck region and facial convexity showed the strongest association with difficult facemask ventilation. Our findings show that digital phenotyping complements traditional clinical assessment, potentially improving airway risk assessment in anaesthesia.

## Supporting information


**Figure S1.** Non‐optimism‐corrected receiver operating characteristic curve of the DIFFMASK score in the study cohort.
**Figure S2.** Optimism‐corrected receiver operating characteristic (ROC) curves of the DIFFMASK score using ß‐coefficients of the original published regression model applied to the MASCAN dataset and optimism‐corrected ROC (95%CI) after refitting.
**Figure S3.** Average three‐dimensional facial meshes generated using the FLAME model for male (left) and female (right) patients in the MASCAN study.

**Appendix S1.** Additional information on anaesthesia induction, airway management and three‐dimensional face scanning.
