# Supplementary material for: Pre‐operative three‐dimensional face scans for predicting difficult facemask ventilation: a prospective development study
Source: Anaesthesia. 2026 Jun 5;81(10):1340–8. doi: 10.1111/anae.70261 (PMC13551139; doi:10.1111/anae.70261)

Figure S1 Non-optimism-corrected receiver operating characteristic curve of the DIFFMASK score in our study cohort (AUROC 0.81)

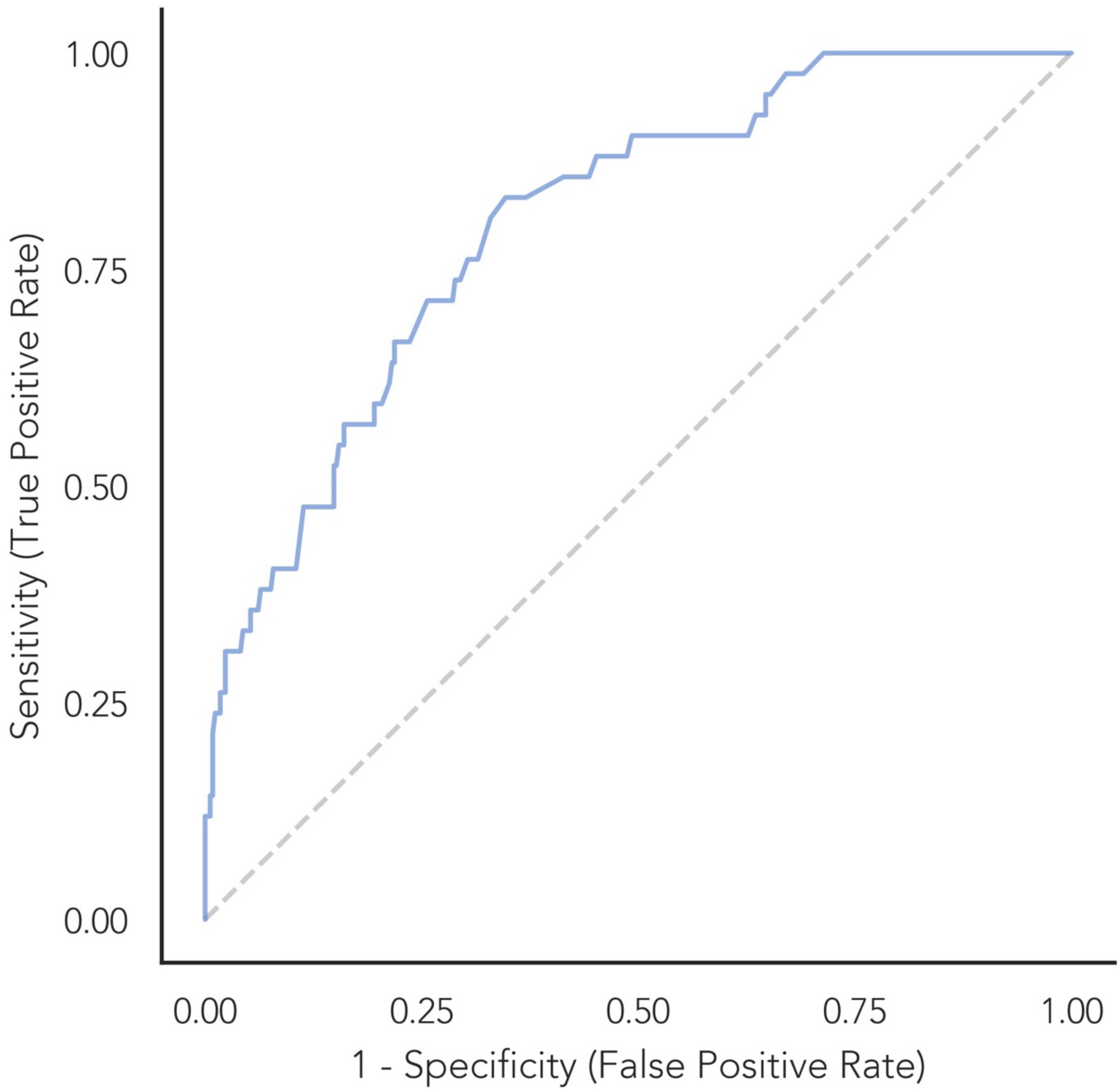

Figure S2 Optimism-corrected receiver operating characteristic (ROC) curves of the DIFFMASK score using ß-coefficients of the original published regression model applied to our MASCAN dataset (blue line) and optimism-corrected ROC (95%CI) after refitting (orange line)

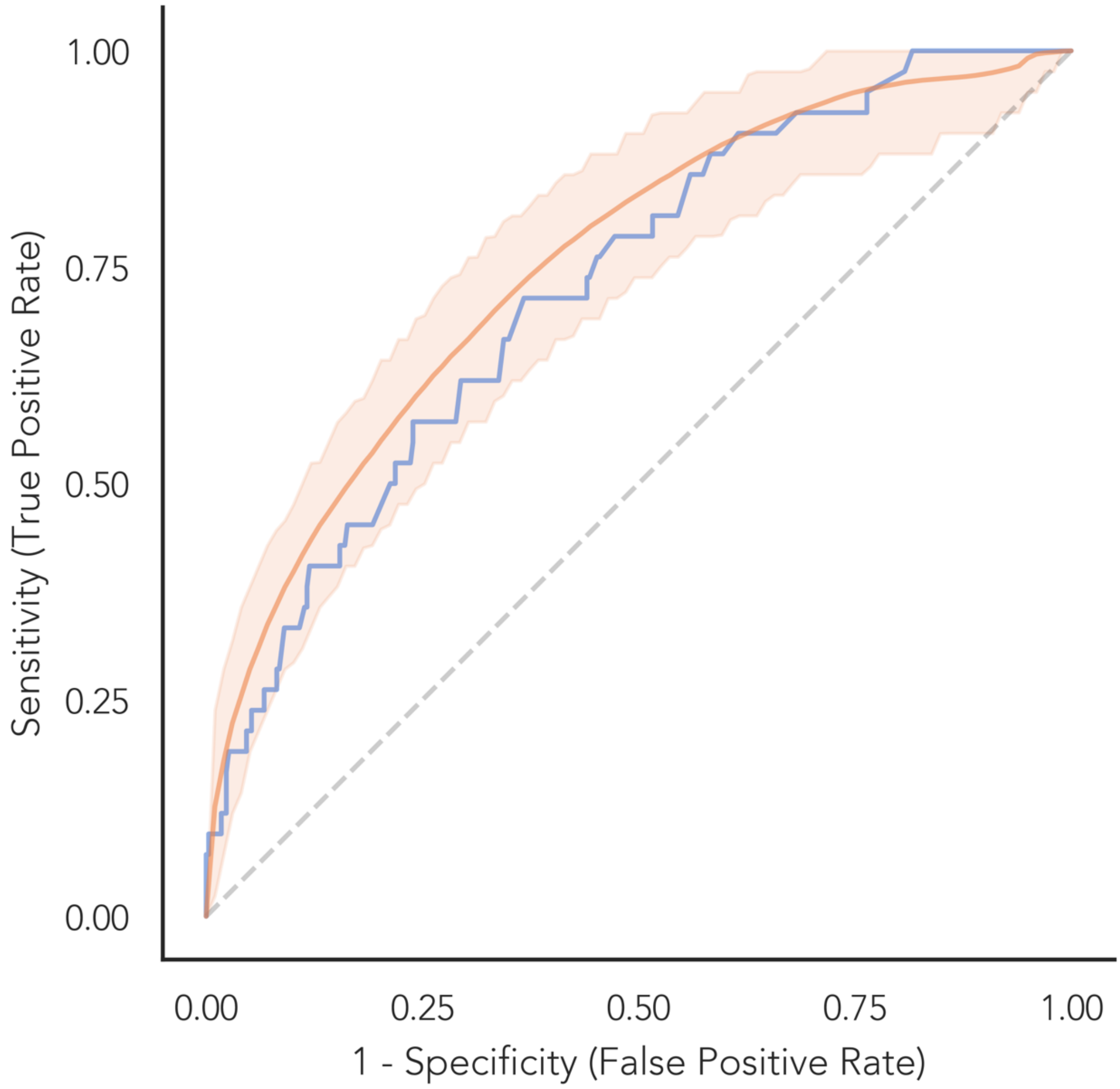

Figure S3 Average three-dimensional facial meshes generated using the FLAME model for male (left) and female (right) participants in the MASCAN study. The displacement between the average fitted mesh for patients with easy and difficult mask ventilation is represented by the superimposed colour gradient. Red regions indicate larger differences, while blue regions indicate smaller differences between these groups.

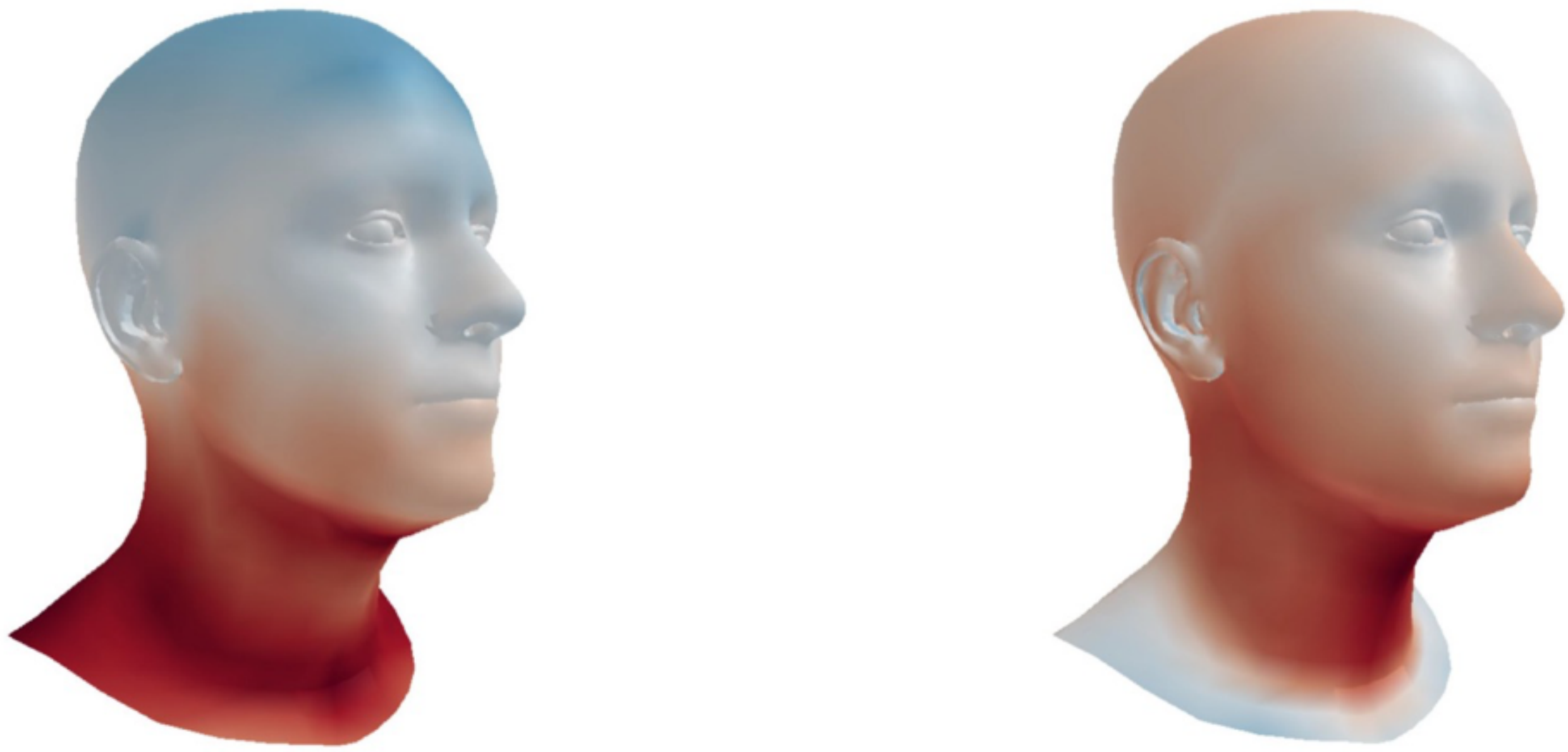

Supplement: Supplementary file 1 [file ANAE-81-1340-s001.pdf]
