# Supplementary material for: Pre‐operative three‐dimensional face scans for predicting difficult facemask ventilation: a prospective development study
Source: Anaesthesia. 2026 Jun 5;81(10):1340–8. doi: 10.1111/anae.70261 (PMC13551139; doi:10.1111/anae.70261)
Supplement: Supplementary file 2 [file ANAE-81-1340-s002.docx]

**Appendix S1 – Additional information on anaesthesia induction, airway management and three-dimensional face scanning (processing, dimensionality reduction and statistical analysis)**

**Anaesthesia induction, facemask ventilation and airway management**

The optimal facemask size was identified by placing different sized masks (Silicone mask, Armstrong Medical, Coleraine, UK) on the patient’s face and selecting the mask that best covered the nose and mouth without excessive overlap or pressure on the eyes, as determined by the airway manager. After the loss of eyelid reflex, facemask ventilation was attempted using pressure-controlled ventilation delivered by a mechanical ventilator (Perseus® A500, Draeger Medical, Lübeck, Germany) with standardised settings: inspiratory pressure 15 cmH_2_O, respiratory rate 10 breaths.min^-1^, PEEP 0 cmH_2_O, I:E ratio 1:2 [1-3]. A fraction of inspired oxygen of 1.0 at 15 l.min^-1^ was delivered. Facemask ventilation was optimised by adjusting head and neck position at the discretion of the airway operator. Rescue manoeuvres such as two-handed grip, oral airway use, jaw thrust, senior consultant anaesthetist taking over or conversion to manual rescue ventilation were performed as needed. Facemask ventilation was evaluated after induction of anaesthesia and before administration of a neuromuscular blocking drug. Rocuronium bromide 0.6–1.2 mg.kg^-1^ predicted body weight (PBW) was then administered to all participants. If the quality of facemask ventilation was not regarded as acceptable by the airway operator, a total dose of rocuronium 1.2 mg.kg^-1^ PBW was ultimately administered, if appropriate. A train-of-four count of 0 (ToFscan®, Draeger Medical, Lübeck, Germany) was checked before tracheal intubation was performed using a direct laryngoscope (Dahlhausen LED Stainless Steel, Cologne, Germany). Airway operators consisted of resident or consultant anaesthetists; their age and duration of professional experience were recorded.

**Three-dimensional face scanning, processing, dimensionality reduction and statistical analysis**

We used the Artec EVA (Artec 3D, Luxembourg) handheld 3D scanner which uses structured light to capture a 3D image and is proven in the medical field [4]. We used the Artec Studio 17 Professional software (Artec 3D, Luxembourg) for data acquisition and post processing. We conducted the examination with participants in an upright sitting position with the back leaning against the chair’s backrest allowing unobstructed access from all sides for the scanning process. We performed the scan by moving the scanner around the participant while continuously checking the process using the real-time fusion option of the software. We maintained the optimal distance between the scanner and participant of 52-88 cm, that is recommended by the manufacturer, by means of the software-integrated distance meter. We required a maximum of 60 seconds for the entire scanning process. We asked patients to remove jewellery and metal accessories from the head and face, if appropriate. Patients tied back long hair when possible; if not, they were asked to at least tuck the hair behind the ears to ensure full visibility of the helix and external auditory canal. We performed a static scan of the patient’s head and neck in a neutral head position, facing forward with the eyes and mouth closed, without clenching the teeth. Independent researchers, who were not involved in the medical treatment of the patients and well trained in the scanning process, performed the 3D face scans using a strict protocol and predefined structured workflow. On the other hand, the entire medical staff involved in patient’s care was blinded to the findings of the 3D face scans and assessment of clinical predictors.

Facial data were stored, analysed and post-processed on a separate workstation. We carefully examined all scans for artefacts, which we removed manually, if required. An independent researcher group (data scientist and specialist for image analysis), who were not involved in the treatment of the patients and not aware of the findings of preoperative risk stratification, clinical course, medical decision making, treatment or outcome of the patients, analysed the facial data at the end of the study.

We processed each scan into a high-quality mesh file using Artec Studio 17 Professional (Artec 3D, Luxembourg), which enabled denoising, cleaning, and smoothing. Each mesh consists of thousands of spatial points, which are both difficult to interpret directly and challenging for statistical modelling due to strong natural correlations across neighbouring points. To circumvent these issues, we leveraged an established representation of facial morphology from outside the clinical domain. Specifically, we automatically located facial landmarks based on the Multi-view Consensus convolutional neural network (CNN) for 3D Facial Landmark Placement by Paulsen et al. [5], which provides pretrained models for the DTU3D configuration (73 landmarks). Then, we automatically fitted each processed face scan to the FLAME (Faces Learned with an Articulated Model and Expressions) model, that is trained on more than 33,000 faces from diverse populations using these landmarks and the 3D mesh [6]. This approach provides a concise, disentangled and low-dimensional latent representation of each face, separating identity, pose, and expression. The identity coefficients encode global facial shape using orthogonal features ordered by their contribution to facial variability. For our analysis, we kept the top 100 identity shape coefficients while discarding pose and expression components, as recommended in the original FLAME publication [6].

The statistical analysis focused on testing whether enriching the standard clinical features with interpretable facial features would lead to a significant better fit in logistic regression models not only explained by the additional model complexity. For feature selection among the facial shape descriptors, we employed the "Adaptive Best Subset Selection" algorithm, which is designed to identify a small, optimally predictive subset of variables likely to yield maximal model accuracy, as described by Zhu et al [7]. Given the nested nature of both models (as the enriched model contains all predictors of the original model plus facial feature variables), we applied the likelihood-ratio test to formally compare model fit while considering the additionally model complexity. Additionally, the area under the receiver operating characteristic (AUROC) with 95% confidence intervals (95% CI) served as metric for discriminative power, enabling assessment of how well the logistic regression model discriminate between patients with and without difficult mask ventilation incidents. For generating the confidence intervals, 1000 bootstrapped datasets of the same size of the original were generated by resampling the original dataset with replacement. The obtained AUROC were optimism-corrected based upon these bootstrapped values using Harrell’s procedure [8]. For all analyses concerning our research questions, a p-value threshold of 0.01 was employed to define statistical significance.

Metric variables were checked for normal distribution by visual inspection of histograms. Sample characteristics are reported and relative frequencies, or median (interquartile range [IQR]). Statistical analyses were performed using Python 3.12, R version 4.3.1 (R Foundation for Statistical Computing, Vienna, Austria) and SPSS statistics version 27 (IBM Inc., Armonk, NY, USA).

**References**

1. Lee MK, Kim KN, Jeong MA, Kim SY, Oh MS, Kwon BS. Facemask ventilation and vocal cord angle following neuromuscular blockade: a prospective observational study(). *Anaesthesia* 2022; **77:** 1010-7. https://doi.org/10.1111/anae.15786.
2. Sato S, Hasegawa M, Okuyama M, et al. Mask Ventilation during Induction of General Anesthesia: Influences of Obstructive Sleep Apnea. *Anesthesiology* 2017; **126:** 28-38. https://doi.org/10.1097/ALN.0000000000001407.
3. Yamasaki T, Komasawa N, Kido H, Minami T. Contribution of the nasal passage to face mask ventilation: a prospective blinded randomized crossover trial. *Can J Anaesth* 2017; **64:** 935-9. https://doi.org/10.1007/s12630-017-0911-3.
4. Modabber A, Peters F, Kniha K, et al. Evaluation of the accuracy of a mobile and a stationary system for three-dimensional facial scanning. *J Craniomaxillofac Surg* 2016; **44**: 1719-24. https://doi.org/10.1016/j.jcms.2016.08.008
5. Paulsen RR, Juhl KA, Haspang TM, Hansen T, Ganz M, Einarsson G. Multi-view consensus CNN for 3D facial landmark placement. In *Jawahar, C., Li, H., Mori, G., Schindler, K. (eds.) Computer Vision – ACCV 2018 Lecture Notes in Computer Science*, vol 11361, *Springer*, *Cham*. https://doi.org/10.1007/978-3-030-20887-5_44
6. Li T, Bolkart T, Black MJ, Li H, Romero J. Learning a model of facial shape and expression from 4D scans. *ACM Transactions on Graph*. (TOG) 2017; **36**: 1-17. https://doi.org/10.1145/3130800.3130813
7. Zhu J, Wang X, Hu L, et al. abess: A Fast Best-Subset Selection Library in Python and R. *Journal of Machine Learning Research* 2022; **23**: 1-7
8. Harrell FE, Jr., Lee KL, Mark DB. Multivariable prognostic models: issues in developing models, evaluating assumptions and adequacy, and measuring and reducing errors. *Stat Med* 1996; **15**: 361-87. https://doi.org/10.1002/(SICI)1097-0258(19960229)15:4<361::AID-SIM168>3.0.CO;2-4
